# A Qualitative Exploration of Self-Kindness and “Treating Oneself” in Contexts of Eating, Weight Regulation and Other Health Behaviors: Implications for Mindfulness-Based Eating Programs

**DOI:** 10.3389/fpsyg.2018.00880

**Published:** 2018-05-30

**Authors:** Helen Egan, Michail Mantzios

**Affiliations:** Department of Psychology, Birmingham City University, Birmingham, United Kingdom

**Keywords:** self-compassion, mindfulness, self-kindness, self-care, eating behaviors, health behaviors

## Abstract

**Background:** Caring for oneself through mindfulness and compassion to improve or enhance health behaviors, and specifically eating behaviors has come to the forefront of scientific inquiry. The experiences and challenges for people in decision making around food within the context of self-kindness for body and mind care have not been previously explored.

**Aims:** This study explored the experiences of eating behaviors in a community sample and examined the understanding of self-kindness and its relationship to eating behaviors and wellbeing of body and mind.

**Methods:** A phenomenological theoretical position was taken; data were collected using individual semi-structured interviews. The sample was twenty-five members of the wider community in the West Midlands in England. The data were analyzed using Braun and Clarke’s (2006) procedural steps for thematic analysis.

**Results:** Two overarching themes were inductively formulated: ‘Thinking about eating’ and ‘Caring for body and mind’. Five themes were constructed: (a) Treat food is exceptional eating, (b) The proof of the pudding is in the planning, (c) Dieting is a dirty word, which are subsumed under Thinking about eating, and (d) Self-kindness is a disavowed abstract noun, and (e) Self-kindness: A rose by any other name; under Caring for body and mind. Participants described a number of ways of treating themselves both with food and with other activities and pleasure in eating was discussed in terms of social aspects of eating rather than food. Two clear contradictions within narratives around eating and health behaviors were shown. Participants largely eschewed the concept of dieting, but described engaging in highly regulated and restrained eating. There was a lack of connection with the notion of self-kindness; although positive eating and exercise health behaviors were undertaken, they were described as necessary self-regulation, not construed as acts of self-kindness.

**Conclusion:** The results suggests a lack of ease in the interpretation of being kind to oneself as a means of improving mental wellbeing, and an inability to relate self-kindness to physical health behaviors. The association of self-kindness with self-indulgence, and the described disconnect between hunger, satiety and pleasure in eating has implications for interpretation of mindful eating scales, practices and interventions.

## Introduction

Self-compassion has become pertinent in contemporary health sciences, and even more so in mediating and predicting physiological health. From randomized controlled trials utilizing self-compassion for smoking cessation (Kelly et al., 2010) and weight regulation (Mantzios and Wilson, 2015a) to predicting health-promoting behaviors and better physical health (Hall et al., 2013; Sirois, 2015; Sirois et al., 2015; Brown et al., 2016; Dunne et al., 2016), self-compassion has become a trait at the forefront of up-to-date inquiries around health and health behavioral change. Compassion has been researched in association with a range of other health behaviors including exercise behavior (Magnus et al., 2010), daily alcohol usage (Brooks et al., 2012) and medical adherence (Brion et al., 2014). Self-compassion has been defined as a mindful awareness of treating oneself with kindness and understanding when faced with adversity, and through realizing that such experiences are common amongst all humans (Neff, 2003b). Neff (2003a,b) described how self-compassion consists of three interrelated components: self-kindness (vs. self-judgment), common humanity (vs. isolation), and mindfulness (vs. over-identification). More recently, increased attention has been given to the role of self-compassion in eating behaviors and weight regulation.

Self-compassion supports healthier eating through negative associations to fat and sugar consumption (Mantzios et al., 2018b), grazing (Mantzios et al., 2018a), motivations to eat palatable foods (Mantzios and Egan, 2018), overeating (Adams and Leary, 2007), eating disorder symptomatology (Taylor et al., 2015), and weight gain (Mantzios et al., 2014). Furthermore, a recent systematic review showed six randomized control trials of self-compassion interventions for nutrition habits, eating behaviors, body weight, and body image (Rahimi-Ardabili et al., 2017). The review suggested that out of six randomized control trials, four investigated weight regulation and proposed positive outcomes in weight regulation (Mantzios and Wilson, 2014; Braun et al., 2016 – Studies 1 and 2; Mantzios and Wilson, 2015a,b).

In more detail, these randomized trials found that participants lost more weight when they were assigned to a mindful self-compassionate program compared to a control group (Mantzios and Wilson, 2015a). Other research attempted to induce self-compassion in a mindfulness context that was more behaviors-specific than mindfulness meditation, whereby Mantzios and Wilson (2014) developed a “mindful concrete construal” diary. The diary was an event-based mindfulness practice to be used with every meal, and aimed at increasing levels of mindfulness and self-compassion when eating. The diary highlighted mindfulness and self-compassion, and assisted participants to observe goals that were visible and achievable in the present moment, while being kind, accepting, and understanding to thoughts and feelings that arose during each meal. The diary was successful in producing similar levels of weight loss to a meditation group, and improved the delay in weight regain. Hussein et al. (2017) found that participants displayed higher levels of mindfulness and compassion in an experiment which assesses whether the diary would work without physically writing in it and by simply considering the questions on the diary. Their hypothesis was that the diary would become more of a priming tool that would assist mindful eating, rather than another activity for people who were having their meal. However, self-compassion remains a trait that requires further research, partially because of the limited evidence, but also as it is uncertain whether (or not) self-compassion (and elements inclusive within the scale such as self-kindness) lead to healthy choices around food, despite the weight loss that has been observed. In fact, some researchers have argued that self-kindness may not be as effective in predicting health behaviors. For example, Mantzios and Egan (2017) suggested that self-kindness should be recommended with caution to people who seek support with health behaviors, as items in the self-compassion scale such as “I’m kind to myself when I’m experiencing suffering” are exposed to an idiographic understanding of the participant, and do not dictate how self-kindness is enacted in everyday life. In effect, the way people display kindness to themselves in times of distress may be to take a walk in nature, or, to eat a family-size packet of cookies.

Mantzios and Egan (2017) further suggested that the association of self-kindness with self-care, and an equilibrium of being kind to our body and mind may be a true reflection of self-compassion (see Neff, 2011) in enhancing health behavior change and health outcomes when they were discussing eating behaviors. They further proposed that the wider separation between psychological and physiological health within the concept of self-kindness, and in effect, of self-compassion, or any other mindfulness-based construct may be problematic. This is not to say that self-kindness is ineffective in health contexts, but that it may moderate positive relationships. While self-kindness displays a positive method of alleviating psychological distress, being kind to yourself does not necessarily mean that both psychological and physiological health are attained simultaneously. The present study explores how people understand and enact self-kindness focusing particularly on eating behaviors and other related health behaviors including exercise and drinking alcohol.

## Materials and Methods

### Participants

Twenty-five participants (males = 10; *M*_age_ = 42.7, *SD* = 15.2) aged between 22 and 73 years from across the West Midlands in the United Kingdom were recruited through opportunity sampling. Five were known to the main researcher and the others were invited by participants or by the wider research team to take part in a research study to explore our understanding of how we show kindness and how this might be related to how and what we eat. The sample displayed an average BMI of 25.4 (SD = 4.5), and scores ranged from 18 to 33. Fifteen participants were White British, seven Irish White, one White Russian, one Chinese-Australian, and one German. Eight participants were educated to post graduate level, seven were graduate level, six were current undergraduates, one had no formal qualifications and three did not disclose their level of educational attainment. Fourteen participants were not happy with their weight, and five reported that they were regularly smoking.

### Semi-Structured Interview

The semi-structured interviews examined participants’ beliefs, thoughts and behaviors around eating to further understand the role of self-kindness and self-care as well as treating oneself through foods. These were examined by focusing on health behaviors associated with eating including lifestyle choices around eating behaviors, alcohol consumption and exercise. Questions included: *If you want to ‘treat’ yourself, what sort of things would you do? ‘Have you ever dieted?* and ‘*Is eating a pleasurable experience for you?’* The impact of self-care for body and mind was explored in line with previous literature suggesting it to be vital in explaining eating and other health behaviors as key factors of reduced wellbeing.

The interviews were conducted by two researchers and took place in private rooms. The interviews lasted between 30 and 90 minutes. All participants volunteered to take part, and participants were provided with pseudonyms to ensure confidentiality. Participants had the ability to withdraw at any time from the research. Ethical approval was obtained by the Business, Law and Social Sciences Ethics Committee of the host University of the researchers.

### Analysis

The data were coded for information about the specific research questions stated in the introduction. Data were analyzed inductively from a contextualist point of view (Braun and Clarke, 2006). The recordings of the interviews were transcribed by the two interviewers using a light version of the Jefferson system (Lapadat and Lindsay, 1999), and a key to transcription conventions is included in **Table 1**. Data familiarization happened throughout data collection and transcription. Coding was performed line by line, with each code providing a label to describe the content of the quote selected. Each code represented something interesting or important about that subdivision of data.

**Table 1:** Transcription conventions.

[⋅]	Mark the start and end of overlapping speech
Underlining	For emphasis
CAPITALS	Speech that is obviously lounder than surrounding speech
(⋅)	A micropause, hearable but too short to measure
##	To indicate long pauses with # symbolizing 1 s
> he said <	greater than and lesser than signs enclose speeded up talk
She wa:anted	Colons show degrees of elongation of the prior sound, the more colons the more elongation, roughly one colon per syllable length

The validity of results were supported once all researchers agreed on a collective interpretation of the data. For this to occur, the researchers presented their initial codes, reflected on similarities and differences between codes, and allowed for revisions based upon researchers’ similar and differing perspectives of the data and the concurrence (or conflict) to the relevant literature. This method of triangulation demonstrated that relevant theories were challenged and integrated to produce a clear understanding on eating behaviors and how they relate to self-kindness, mindfulness constructs and self-care.

Once codes were finalized, the researchers jointly generated the themes by classifying each code into groups of codes. Assessing the themes in support of the data and in line with the overarching theoretical perspectives enabled the researchers in defining what each theme stood for, and what was significant about the themes. Specific participant quotes were chosen to illustrate the main themes and subthemes. These quotes are presented in the Results section.

## Results

Thematic analysis identified five themes which were constructed and developed from the data during analysis (**Table 2**). The first, ‘Treat food is exceptional eating’ explores participants’ descriptions and explanations of how eating treat food is different from usual eating and incorporates what and how often treat food is eaten. Theme two, ‘The proof of the pudding is in the planning’ highlights the significance and prominence of planning and preparation of food as a more central aspect of pleasure in eating than the act of eating as such. In theme three ‘Dieting is a dirty word’ the reluctance of participants to explicitly label restrictive and regulatory eating and exercise behaviors as dieting is explored. Theme four ‘Self-kindness is a disavowed abstract noun’ explores participants’ lack of identification with the term self-kindness, with many showing a clear discomfort and distaste for the term. In the final theme ‘Self-kindness: A rose by any other name’ discusses the ways in which participants enact self-care to their mind and/or body whilst simultaneously rejecting the notion that they are being kind to themselves.

**Table 2:** Development of Themes from Codes.

Thinking about eating			Caring for body and mind	
1	2	3	4	5

**Treat food is exceptional eating**	**The proof (pleasure) of the pudding is in the planning**	**Dieting is a dirty word**	**Self-kindness is a disavowed abstract noun**	**Self-kindness: A rose by any other name**
Sugar and Fat treats	Social eating	Self-regulation	Self-kindness is self-indulgent (an optional extra)	Time out is a treat
Alcohol is a treat	Food as fuel	Dieting is hard	Exercise is not self-kindness	Exercise is caring for my body
Treat frequency	Self-licensing	Not dieting, watching what I eat	Dieting is not self-kindness	Exercise is caring for my mind
Treat food is more than just fuel	Dwelling/not dwelling	Cues to lose weight	Food treats are not self-kindness	Good for me/not good for me
	Pleasure in food	Exercise as weight control		Treat food is not kind to my body
	Pleasure in food preparation			

### “Treat Food Is Exceptional Eating”

The majority of participants described using food as a way to treat themselves with only a very few rejecting food as a potential treat. The treat element of this eating behavior was constructed in a number of different ways, with the common understanding that the eating of treat food was in some way different from usual eating behaviors. For some people it was specific foods that were treats, and these were frequently high fat and sugar foods:

**Michael** *Probably something naughty in the food department, you know, something packed with fat and sugar....*

**Lucy** *I’d have a bag of chips and a magnum.*

These foods were considered treats not only because of their ‘unhealthy’ nature, but also as they were not eaten very often, and in fact, the specificity of the food as a treat was dependent on infrequent consumption:

**Violet** *Chocolate is a treat # it’s not an everyday aspect of my diet # I don’t eat it everyday (.) I don’t even eat it every week.*

**Carol** *Well, we used to use food as a treat but now it’s kind of an everyday, if I haven’t had my chocolate by the afternoon I think hmmm, where’s my chocolate? So maybe it’s not so much a treat anymore...*

A small number of participants described using alcohol as a treat, with participants rationalizing in the same way as for treat food, that for alcohol to be a treat it has to be different from normal consumption, for example, more expensive or better quality than usual:

**Sam** *Have a glass of wine, buy more expensive bottle than I would do normally.*

**Gavin** *But a good bottle of wine # would be a regular treat.*

There was a degree of self-consciousness or self-judgment about citing alcohol as a treat and this was evident across the data when people talked about food or alcohol as a treat being not as ‘worthy’ as other treats (See theme 5):

**Lucy** *Yeah. I’d like to think that I could say to you that I’d have a nice long bath and I’d read a good book but I’d be lying.*

**Rory** *That’s probably the # that’s probably the # biggest treat ## that’s sad that (.) alcohol.*

**Michael** *As a treat? I suppose a beer. That’s, you know, a bit trivial, but beer.*

For a number of people the treat element of the food was about quality and about the time and the effort that goes into preparing the food, rather than about specific foods. Participants talked about treating themselves with food in terms of cooking something different from usual, and of spending more time in planning and cooking than they would usually do:

**Sam** *But not routinely, no. Unless you would say, I want to cook a nice dinner for everybody, not just fuel.*

**Kate** *I think ummm surprisingly enough, when I want to treat myself I like to eat nice food… not necessarily a shop bought….. more I like to cook for myself……something I can be proud of and makes you feel good.*

The understanding here is that food and eating is often mundane and based on convenience (time) rather than something that people expend time and effort in engaging. The relationship between time and effort and pleasure in eating behaviors is explored further in the second theme.

### “The Proof of the Pudding Is in the Planning”

When participants were asked whether eating was a pleasurable experience for them, responses focused on factors that were associated with eating, but were not specifically about the actual behavioral enactment of eating. Planning and preparation increased both the anticipation of pleasure, and the experience of pleasure in eating.

**Bob** *I suppose a lot of the pleasure is the anticipation of the food, um so like, you might buy something in the shop or cook something really nice but actually it’s more about the anticipation, ‘ooh I’m going to have a nice curry/pizza’ and them sometimes I feel when you’re eating it you almost don’t appreciate it as much as you should do, you just like scoff it and that’s it.*

**Violet** *I enjoy preparing food # e:erm:m ## >I enjoy shopping for food I enjoy preparing< # I love cooking for other people # e:erm:m # I like creating colorful dishes ## when I make myself a salad then it becomes a work of art on the plate # so it’s about ### food being something that’s # creating ###.*

**Jane** *I like thinking about food, I like planning an exciting meal. Socializing with friends, which is definitely a treat, frequently involves people coming round to our house and having dinner.*

While for some, pleasure was increased when they were engaged with preparation, for others, or for the same people at other times, the alleviation of the need to plan and prepare increased the pleasure in eating. In the first extract Rory is clear that it is not about what the food actually is, but the ability to eat without having to prepare that is important:

**Rory** *It invo:olves me not doing something # it doesn’t ## it’s not like ## chocolate # o:or ## steak ## it doesn’t matter (.) it’s someone else doing the wo:ork for me.*

**Lucy** *Eating socially with people is completely different because then there is the element that you would choose something if you go out for a meal you would choose something and that’s where the treat comes in because you would think ‘ooh yes I’ll have that’ because there’s no way I would cook it.*

The significance of eating as a social activity, and its association with increased pleasure was apparent across many participants. Social eating was understood to change the nature of standard eating in ways concerned with factors other than the actual food. Increased food choice, which was linked to eating food without the need for preparation was the only food factor which was cited as increasing pleasure in eating by participants:

**Cameron** *E:erm:m # two parts actually # one is # having food # well one is (.) having food you ca:an’t cook yourself….it’s part explo:oring new # new things ## so (.) trying new restaurants.*

**Fay** ‘*Cause you can pick off a menu # pick what you actually fancied and wanted.*

Other elements of increased pleasure were focused on the social rather than the eating element of social eating; who people were with, the setting of the meal, the atmosphere, and the luxury of prolonged periods of time spent over a meal were all considered important and relevant factors in increasing the pleasure in the experience:

**Lucy** *Yeah, it totally alters whatever I eat if I am eating it socially. It’s the conversation, the company. I think food then takes on a completely different meaning. And then, I don’t care if the cheesecake has 10,000 calories. I’m not living like this everyday so I wouldn’t spoil that occasion by saying well you know can I have the no dressing on the salad or whatever.*

**Carol** *Yeah what goes with it like the thought, like if somebody has cooked it, or if it is, you go out for dinner if it is a bit of a nice, well if it is a nice restaurant, a bit of a nice atmosphere sort of thing.*

**Violet** *We were in France recently with friends (.) and lunch was a two hour # two course # dinner is like four hour # five courses [laughs] # and it’s just that whole talking (.) socializing # and eating at the same time # I love that.*

There were many instances of people talking about food and eating in ways that demonstrated a rather bleak experience of eating, when food was considered to be little more than fuel. For a few this was an overall feeling about food and eating: (Both of the participants quoted below reported that they were happy with their weight and currently self-regulated their weight, but were not dieting).

**Lucy** *No. It’s something that stops you falling over. So that’s where you have to, in some ways be more careful, because you can eat a very unbalanced diet; if all you want is just something that will stop you from falling over it can be anything.*

**Theo** *I think my whole attitude toward food is # I need it.*

Whilst these participants were in the minority, many other participants described times when food was just fuel, this kind of eating could be described as functional, or even mindless and was never associated with pleasure:

**Carol** *But sometimes I do just use it as a, I need energy I’m the type of person that can’t even have a cup of tea in the morning unless I’ve had some food. So that’s a case of eating something before you can even get on with your day. And lunch I just sort of grab what’s going.*

**Fay** *Yeah (.) functional food # I don’t li:ike functional food # it ha:as to be interesting # and that’s why I think cooking is an effort for me # cause I don’t just eat pla:ain # dinners.*

**Jan** *Sometimes you’re just starving aren’t you and you just, starving, so you eat something just ‘cause you’re busy and you haven’t got time to sit down and enjoy a meal. So you end up eating on the run.*

The eating described above is eating in response to hunger cues, which is part of primitive descriptions of mindful eating, but it is far from mindful eating due to the non-existence of other elements such as focusing on the present meal and getting more pleasure than usual. When food is eaten without any thought and engagement it cannot be described as mindful eating, but rather a mindless association with food that results in a lack of pleasure and residual negative feelings about the food consumed:

**Jan** *But you get mad with yourself if you just, pick something up, and eat it. Yeah, you just pick it up and you just do, and you’ve eaten it and you didn’t actually enjoy it that much, and you think. Damn it. That just cost me 12 points that did…has... and I didn’t even enjoy it…. You get annoyed.*

Interestingly, like social eating, the feelings of pleasure in eating restricted foods was similarly associated with factors other than the actual consumption of the food. While a lack of conscious decision making around eating food decreased pleasure, pleasure was increased when there was a decision made that food is going to be eaten (self-licensing) and this remained even when the food eaten was self-considered to be excessive or breaking usual eating ‘rules’:

**Jane** *If you’ve made a conscious decision, like say you went to a function and you, you know you’re going to be drinking and eating buffet food which is rubbish for dieting then you just have to accept that and that’s, that’s what’s going to happen. And you don’t get mad with yourself.*

**Gavin** *o:or if I think tonight (.) it’s been a long whole week # I’m going to cook that duck # bottle of red # and then we’ll have some chocolate # I don’t give a shit.*

Conscious decision making and self-licensing not only increased reported pleasure in the eating, but was also aligned to decreased feelings of self-judgment and dwelling after consumption:

**Jan** *I don’t really torture myself or anything. Just, if you break it, you break it. And tomorrow’s another day*.

**Kate** *I feel a bit guilty for eating. I do speak to myself and say it’s a one off, at the end of the day if you enjoyed it doesn’t really matter that much in the grand scheme of things.*

**Michael** *I’ve got broad shoulders. I sort of say hey ho admit it and just move on, you know, I’ll get it right next time.*

The above data tells a clear story of highly regulated and often restricted eating behaviors, however, none of the participants described themselves as actively dieting and this seeming contradiction is discussed in theme three next.

### “Dieting Is a Dirty Word”

In response to the question ‘Have you ever dieted?’ almost all participants responded negatively, eschewing the concept of dieting. Nevertheless, they immediately followed with explanations and examples of eating behaviors in which they engaged when they felt that they needed to lose weight, including cutting down on calories, portion control, and restricting certain foods, all of which could be considered as dieting behaviors:

**Jane** *I don’t think I would ever say I have dieted. I have definitely over times tried to cut down the amount of calories I consume. I wouldn’t call it dieting cause I’ve never followed a specific diet, I’ve never counted calories, I’ve never weighed food. I’m just very much aware of how much I’m putting in on a regular basis.*

**Bella** *But I’ve never done anything else # just cut do:own.*

**Michael** *I think I try if I feel I’m putting weight on then I will cut back cause that’s the point where I will watch what I’m eating. But I don’t change what I’m eating it’s usually the quantity. I’ll cut down on my chocolate and my sugars and my puddings.*

**Jake** *Yeah it’s not my thing, I’ve never tried those. Mostly I just eat less. I don’t skip meals, I just have smaller portions.*

**Edward** *No # it would just be about portion control.*

Dieting was then considered to be something that you ‘did’ to yourself, and entailed following a regime of eating for a certain period of time that was portrayed as somehow different from usual eating behaviors. It was an undesirable activity not necessarily because it entailed restriction, but rather because it was perceived as not healthy or sensible:

**Kate** *umm personally I don’t really believe in um the celebrity diets that you read about in magazines. I like a balance of healthy food, in not too high a quantity, and exercise.*

**Rory** *Rather than dieting # albiet # I’m trying to make # sensible choices with food ## not blow out every night # say no sometimes.*

**Ella** *I don’t start a diet as I guess I’m in some way always sensible, I don’t ever undergo fad diets, I just constantly eat not as much as I would like.*

For those participants who had in the past engaged in active dieting, they described the experience as unhelpful and self-defeating, and definitely not an act of self-kindness but also described current self-regulatory eating behaviors as positive and productive in terms of weight maintenance.

**Violet** *I’ve tried them # I tried calorie counting diets # tried milkshake diets ## I think the sheer word of diet is just di:ie (.) with a t at the end of it # and not a good t ##....If I go into calorie counting # o:or depriva:ation of any sort # then it provokes a (.) pissed off # fucked off # angry reaction ## and when I get emotional I want to eat ## it’s self-defeating.*

**Kate** *I have in the past done low calorie and calorie counting but they have been in general for quite short periods and I don’t think they really work.*

**Fay** *I re:eally don’t like the way I am # but # it takes a considerable amount of effort # and time # that I don’t seem to have ## to do the dieting.*

Watching what I eat is perceived here as engaging in behaviors for the purpose of losing weight usually over a discreet period of time. Participants also talked about ways in which they engaged in weight regulation to maintain current weight and avoid weight regain. This was different from watching what they ate in so far as it was a continuous activity, rather than in response to weight gain and the desire to lose weight.

**Theo** *Well if I can eat healthy I do:o ## if there’s a healthier choice I’ll probably make that choice #.*

**Gavin** *And then # for most part # I’ll wait till dinner time # and try not to snack too much # but that’s become conscious now # whereas befo:ore it’d be (.) I’m hungry (.) bowl of cereal # I’m hungry (.) right chocolate.*

**Ella** *Well yes I’d eat less the next day or do some exercise, I think for me it’s just a continual awareness in what I’m eating.*

Exercise was also suggested to be a big part of weight regulation and was frequently communicated as an alternative to dieting for the purposes of both losing and maintaining weight:

**Jane** *But I do also use exercise, as a way of, meaning that I can still eat some of those things and possibly, lose a little bit of weight.*

**Theo** *I’ve never # ever been on a diet # and ###.hhh # I don’t know # I’ve always done a lot of sports # it’s never been an issue.*

**Rory** *I’m trying to be active (.) and healthy # so I’m cycling to work (.) and I’ll cycle the long way # in order to get (.) a bit mo:ore.*

**Fay** *Yeah # so I think I prefer ## eating what I want and exercising mo:ore # than not exercising (.) and eating a strict diet.*

Exercise was seen as a positive activity for the most part, particularly when there was a secondary gain such as playing team sports or as a means of transportation (e.g., cycling and walking). It was perceived by participants not as a way of being kind to themselves, but rather as something that was necessary and this distinction is further explored in the next theme.

### Self-Kindness Is a Disavowed Abstract Noun

Participants were uncomfortable with considering the concept of being kind to themselves, not only strongly rejecting self-kindness as a normative way of being, but rejecting it as a desirable way for them to be and expressing that they found it difficult to act with self-kindness:

**Sam** *No, No, I don’t think so I don’t think of being kind to myself.*

**Gavin** *self-ki:indness # I’m # not ## that # good # I struggle with that.*

**Jake** *I don’t think I should ever be kind to myself because that sounds like it isn’t going to drive me to do anything.*

Self-kindness was articulated negatively as an ‘indulgent’ optional extra, only two people spoke of self-kindness as important and necessary and both of them talked about having a strong religious faith. When asked whether exercise was a way of being kind to oneself, there was a clear message that it was not, participants used the term necessary as an opposite to self-kindness:

**Theo** *hhh # well the things I do for me # large part # sports… But I don’t just do them because I’m being ki:ind to myself # I think it’s just a necessity # I ne:eed that.*

**Bella** *# E:er:r ## I’ve never thought about it (exercise) in that sense ## I would say it’s a necessity.*

This conflict between necessity and self-kindness was also seen when healthy eating was discussed, which was considered as something that should be done, rather than an act of self-kindness:

**Cameron** *I wouldn’t necessarily think that’s being kind to myself (.) I think it’s more ## that’s what you should be doing.….. but it could be perceived as an act of kindness to myself because I’m happier by do:oing those things.*

**Sam** *Umm (.) I don’t know. I wouldn’t use that word. I wouldn’t use the word kindness. I suppose it is doing something for myself that I wanted to do. I have restricted my food because I wanted to lose weight.*

Self-kindness then was conceptualized in a very different way from how it is within academic literature, with clear implications for mindful practices, and this is explored in the next theme.

### Self-Kindness: A Rose by Any Other Name

A lack of identification with self-kindness as a way of caring for oneself was apparent across the data, but that is not to say that people were not engaging in self-caring ways, rather that they did not see this as being kind, and this further highlights the apparent and misconstrued associated negative connotations of self-kindness:

**Cameron** *So:o ## well I think there’s a difference between (.) being ki:ind to yourself (.) and looking after yourself.*

People were very clear and eloquent about exercise being a way of looking after themselves, benefiting both their body and their mind. The ways they talked about the positive outcomes are very much aligned with self-kindness and self-care:

**Jane** *I always feel better when I’ve been for one (a walk) I do always come back feeling happier. My mind is clear, I’ve probably got endorphins rushing round my body, I’m happy.*

**Theo** *No # it’s something that keeps you healthy # keeps you fit ## a:and # gets rid of some of the # stress ## that you have from other parts of your life.*

**Cameron** *I know # I’m a much ha:appier person when I exerci:ise ## I eat well # I’m not stressed # them kind of things.*

While people were uncomfortable and unwilling to talk about ways they were displaying kindness to themselves, they were happy to discuss ways in which they treated themselves when this treating did not include food and drink. Such treats engendered less self-consciousness and self-judgment, and were considered more worthy ways of treating oneself. Taking time out from everyday life was an oft mentioned treat:

**James** *e:erm:m I’d spend time doing nothing.*

**Jake** *A treat to me would be things like taking my laptop and going and hiding in a coffee shop for three hour. Where I will relieve myself of the other burdens of life and I will forget I have a baby and will throw myself into working.*

**Rory** *Yeah (.) it’s me time ## so that # that kind of thing # I suppose at times it’s just giving yourself an excuse to get off the treadmill ## it’s # it’s just stopping (.) and saying (.) no: # I deserve something nice.*

**Edward** *Yeah # yeah ## a treat would be ## time to yourself # doing what you want to do (.) ra:ather than ### you know (.) >busy busy busy<.*

**Marian** *Stay in bed # read ## eat when I feel like it.*

Participants often framed these acts of self-care and self-kindness as something that they had ‘earned’, or as a reward, for example at the end of a hard day. This is perhaps a way to enable them to feel less uncomfortable about ‘indulging’ in self-kindness.

## Discussion

This research identified two overarching themes ‘Thinking about eating’ and ‘Caring for body and mind’ and five main themes: (a) Treat food is exceptional eating, (b) The proof (pleasure) of the pudding is in the planning, (c) Dieting is a dirty word, (d) Self-kindness is a disavowed abstract noun, and (e) Self-kindness: A rose by any other name, which will be explored with relevant literature next.

The principal notion around the first three themes was how much thought was going into everyday eating and consumption of foods. ‘Treat food is exceptional eating’ suggested an overall consumption of high-calorific foods as treats, and the fall-back on foods that are primarily responsible for the current obesity epidemic (e.g., Ruanpeng et al., 2017). Alcohol was also a primary treat that existed in accordance, or as a substitute for, treating oneself (e.g., O’Donovan et al., 2018). Participants identified the nature and enactment behind the whole idea of having a treat, which had some common elements, namely, that they are infrequent, special and are pleasurable, and were either instant (high fat and sugar foods) or took time and effort to prepare, which reflect the value, quality and cost of the treat.

Some participants talked about food and eating in terms of necessity rather than pleasure, and for them the focus of food was very much around the instant nature of foods, and a description of ‘fuelling-up with energy’ and ‘staying alive’. This was associated with an enhanced ability to observe and respond to hunger and satiety, which are primary examples within the literature of mindful eating (Bays, 2017). But then again, the description from participants conveyed a dispassionate, chore-like activity, which is fundamentally contradicting previous literature and mindful eating (e.g., Hong et al., 2011).

This notion of ‘food as more than fuel’ made its presence in ‘The proof of the pudding is in the planning’ theme, where pleasure in food was associated in concurrence with the social aspect of eating, as well as the preparation and nature of foods. The ability and privilege to take time in preparing a meal, as well as the enhanced quality of home cooked meals appear to be sensory elements of increased pleasure. Furthermore, to socialize around the table enhanced pleasure, potentially through an environmental force of shifting the primary focus on the meal itself, which fundamentally can be seen as a mindful eating paradigm. Likewise, the ability to make a mindful decision around food appear to be a primary focus of enhancing pleasure (or discouraging displeasure). Giving oneself permission to indulge in their favorite foods, or, to eat freely at the party meant that participants may have given themselves a ‘free pass’ (otherwise known as self-licensing – see Witt Huberts et al., 2012). Special and infrequent occasions enable further mindfulness elements to emerge (such as not dwelling, feeling guilty or ashamed) which reinforce a non-judgmental and self-compassionate stance (see Kabat-Zinn, 1990; Neff, 2003b). Although unhealthy, self-licensing was not mentioned over the occurrence of habits, but rather over the infrequency of treating oneself. In many respects, treating oneself with food displayed a non-risk factor for obesity.

The theme ‘Dieting is a dirty word’ explored how people articulated their eating behaviors in terms of regulation rather than dieting. Dieting was a non-engaging topic of discussion, while most of the participants described that they were watching what they eat. This entailed a constant self-regulatory agent, which dictates the inclusion of relevant literature around restrained eaters. When dieting has been associated with habitual overeating (Hays and Roberts, 2008), eating disinhibition (Westenhoefer et al., 1994), and as being a forceful agent of the hedonic drive (Papies et al., 2007), data suggests that labeling dieting as restraint is perceived as a healthier and more effective means of weight regulation. Participants also described using exercise in self-regulatory way to compensate for food consumption. However, exercise in association with restraint eating appears to be another risk factor for obesity (see Ogden et al., 2017). Despite the attempts by National Health Trusts in increasing healthier eating, the ability to self-regulate through restriction and physical activity appears to be problematic, as restrictive eating appears to be at the forefront in predicting weight-loss failure and obesity. However, this may relate to how exercise is being used, particularly in terms of estimating the amount of physical exertion needed to burn sufficient calories for food eaten.

“Self-kindness: A rose by any other name” suggested that exercise was being used as an element of self-care, but some participants referred to physiological health, while others referred to mental health. The divide between self-care and the association with either body or mind was recently discussed when exploring elements of self-kindness (Egan and Mantzios, unpublished). Self-kindness, which is a component within the theorisation and measurement of self-compassion was suggested to be a vital element within health behaviors, especially when an equilibrium of self-care is reached between body and mind (Mantzios and Egan, 2017). Similarly, participants in the present research expressed notions of doing something for their body and physiological health, while others noted how it improved their mental health and reinforced the idea of taking some time for oneself. Interestingly, taking time out, was seen as a treat, and did not relate to any unhealthy behaviors, whether it related to food or not. Within the equilibrium of body and mind, taking time out for a bath or a hike is a neutral activity of self-care, which at the same time does not disadvantage either psychological or physiological health.

The association of health behaviors to self-kindness appeared to be rather difficult, which suggested the theme ‘Self-kindness is a disavowed abstract noun.” While Mantzios and Egan (2017) theorized an association between “treating oneself” with food and “self-kindness,” which may have implications for health and weight regulation, participants in this research suggested that self-kindness relates to self-indulgence, but not to treating oneself to food. Self-kindness was advocated as having no relation to dieting and exercise. Despite the non-association of self-kindness and “treat foods,” self-kindness was described as self-indulgent (cf. Neff, 2011; Mantzios and Egan, 2017), which is problematic in many respects. The ability to be kind to others is more easily conceived and enacted than kindness to oneself, and has been noted in other literature (Mantzios and Wilson, 2015b), and perceptions of kindness and self-kindness, as well as compassion and self-compassion are explored in research that is describing problems and individual differences in what those constructs mean for the general public (Egan and Mantzios, unpublished). The perceptions of people in the current research where they are disassociating self-kindness from any healthy behaviors, and are aligning self-kindness to self-indulgence, suggests regulating food intake and asking people to be kind to themselves may be mismatched and misconceived, unless there is clarity of body and mind self-care as suggested in previous literature.

Limitations that should be taken into consideration include the Caucasian, educated, and geographically contained population that was used for this research. Future research should attempt to recruit a more culturally diverse sample, and possibly disadvantaged individuals who are struggling financially and/or are seeking work. Perceptions and interpretations of kindness and treats may differ from our current population, and further explorations are required to draw clear conclusions.

## Conclusion

Practical applications of mindfulness, self-compassion, and mindful eating require further considerations and refinement to apply to health behaviors and eating. First, the association of self-kindness and self-indulgence creates difficulties in integrating self-kindness into practical programs for weight regulation. Similar connotations could be made when we reflect on mindfulness and mindful eating programs when we explore the element of non-judgment. Second, mindful eating scales may be misconstrued by researchers when interpreting elements of mindful eating, such as feelings of satiety and hunger, as well as pleasure in eating favorite foods. Third, treat foods, which may be unhealthy, are distinct from any notion of habitual consumption and entail infrequent, instant and expensive food endeavors that are more commonly seen as foods that are consumed mindfully. The choice of food may dictate the way we eat, rather than our general demeanor around food. Licensing oneself to have cheese-puffs or an expensive chocolate bar (purchased from a chocolate boutique) may be the difference in eating mindlessly or mindfully, and being kind to oneself certainly aligns to the mindful consumption of a chocolate bar.

## Author Contributions

HE and MM contributed equally to the conception and writing of this manuscript.

## Conflict of Interest Statement

The authors declare that the research was conducted in the absence of any commercial or financial relationships that could be construed as a potential conflict of interest.
